# Integrated Analysis of Single-Cell and Bulk RNA Data Reveals Complexity and Significance of the Melanoma Interactome

**DOI:** 10.3390/cancers17010148

**Published:** 2025-01-05

**Authors:** Michael J. Diaz, Jasmine T. Tran, Arthur M. Samia, Mahtab Forouzandeh, Jane M. Grant-Kels, Marjorie E. Montanez-Wiscovich

**Affiliations:** 1 College of Medicine, University of Florida, Gainesville, FL 32610, USA; 2 School of Medicine, Indiana University, Indianapolis, IN 46202, USA; jasmtran@iu.edu; 3 Department of Dermatology, University of Florida College of Medicine, Gainesville, FL 32606, USA; asamia@dermatology.med.ufl.edu (A.M.S.);; 4 Department of Dermatology, University of Connecticut School of Medicine, Farmington, CT 06032, USA

**Keywords:** melanoma, gene expression, scRNA, network modules, cell-cell communications, ligand-receptor, crosstalk

## Abstract

**Background:** Despite significant strides in anti-melanoma therapies, resistance and recurrence remain major challenges. A deeper understanding of the underlying biology of these challenges is necessary for developing more effective treatment paradigms. **Methods:** Melanoma single-cell data were retrieved from the Broad Single Cell Portal (SCP11). High-dimensional weighted gene co-expression network analysis (hdWGCNA), CellChat, and ligand-receptor relative crosstalk (RC) scoring were employed to evaluate intercellular and intracellular signaling. The prognostic value of key regulatory genes was assessed via Kaplan-Meier (KM) survival analysis using the ‘SKCM-TCGA’ dataset. **Results:** Twenty-seven (27) gene co-expression modules were identified via hdWGCNA. Notable findings include NRAS Q61L melanomas being enriched for modules involving C19orf10 and ARF4, while BRAF V600E melanomas were enriched for modules involving *ALAS1* and *MYO1B*. Additionally, CellChat analysis highlighted several dominant signaling pathways, namely MHC-II, CD99, and Collagen-receptor signaling, with numerous significant ligand-receptor interactions from melanocytes, including CD99-CD99 communications with cancer-associated fibroblasts, endothelial cells, NK cells, and T-cells. KM analysis revealed that higher expression of *SELL*, *BTLA*, *IL2RG*, *PDGFA*, *CLDN11*, *ITGB3*, and *SPN* improved overall survival, while higher *FGF5* expression correlated with worse survival. Protein-protein interaction network analysis further indicated significant interconnectivity among the identified prognostic genes. **Conclusions:** Overall, these insights underscore critical immune interactions and potential therapeutic targets to combat melanoma resistance, paving the way for more personalized and effective treatment strategies.

## Simple Summary

Melanoma is an aggressive malignancy defined by significant intratumoral heterogeneity, driving its capacity for therapeutic resistance and recurrence. This study adopts a systems-level approach to dissect the melanoma microenvironment, focusing on intricate interactions between malignant cells and immune infiltrates. We identified critical regulatory networks and intercellular communication pathways that appear to influence disease progression. These findings highlight the dynamic interplay between tumor-intrinsic factors and the surrounding microenvironment, revealing potential mechanisms underlying immune evasion and therapy resistance. By mapping these complex interactions, the present study builds on our foundation for precision-based therapeutic strategies tailored to the unique biological landscape of melanoma, offering promise for improved clinical outcomes.

## 1. Introduction

Melanoma is one of the deadliest cutaneous malignancies. From 1975 to 2021, melanoma incidence surged by over 320% [1]. According to the National Cancer Institute, more than one million Americans are actively living with melanoma, and current estimates suggest nearly 20 persons die from melanoma every day in the United States [2]. Over the past decade, significant advancements in targeted therapies and immunotherapies, such as BRAF/MEK inhibitors and immune checkpoint inhibitors, have markedly improved survival rates. However, treatment resistance (innate and acquired) and recurrence remain persistent challenges, partly driven by the remarkable intratumoral heterogeneity of melanoma [3,4,5]. This heterogeneity, arising from melanoma cell plasticity and the complex interplay with diverse stromal and immune cells, contributes to variable patient outcomes and remains a critical focus of ongoing research.

In silico analysis of RNA sequencing data has opened new avenues for studying melanoma by revealing complex molecular interactions at unprecedented depth. Transcriptomic analysis has helped uncover critical ligand-receptor interactions [6,7], metabolic pathways [8,9], and predictive gene expression signatures related to melanoma growth and progression [10,11]. Single-cell sequencing, specifically, has lent to highly granular insights into the dynamic plasticity of melanoma cells, showcasing their ability to transition between differentiated and dedifferentiated states, impacting invasiveness and treatment responsiveness.

Despite these technologies, efforts to comprehensively map regulatory genes and cell-to-cell signaling networks in melanoma at single-cell resolution remain limited. The present study aims to address this gap via a detailed study of the melanoma interactome, potentially invigorating future dermatogenomics research and the development of personalized therapeutic strategies.

## 2. Methods

### 2.1. Overview of Datasets

Single-cell RNA (scRNA) sequencing data representing metastatic melanoma was retrieved from Tirosh et al.’s 2016 study [12]. The corresponding expression data is maintained by the Broad Institute’s Single Cell Portal (SCP11), which includes the expression profiles of 4645 single cells isolated from 19 patients. This data is composed of malignant, immune, stromal, and endothelial cells. No patient identifiers are included in this dataset. The present analysis was restricted to genotyped malignant cells to minimize the influence of heterogeneous cell populations.

To assess the potential interplay between levels of identified regulatory genes and patient prognosis, bulk cutaneous melanoma gene expression data and corresponding survival data were retrieved from the Broad GDAC Firehose portal (http://firebrowse.org/?cohort=SKCM, accessed on 1 April 2024).

### 2.2. Identification of Gene Co-Expression Networks via hdWGCNA Applied to scRNA Data

Weighted gene co-expression networks within the single-cell transcriptome expression data were generated using the R package ‘hdWGCNA’ v0.3.00 [13]. For WGCNA input, only genes expressed in at least 5% of cells were retained for analysis. Inferred cell types were retrieved as a mixture of annotations provided by the original dataset authors and, when unavailable, by SingleR (cells < 100 transcripts ignored). Metacells were constructed by cell type and sample. Co-expression networks were constructed at a soft-thresholding power of 8 on the melanocyte expression set. Hub genes, defined here as the top 10 genes based on the degree of within-network connectivity (numerized by kME), were resolved from each generated network module using the GetHubGenes function. Gene scoring was performed using the UCell algorithm (n_genes = 25) [14].

### 2.3. Identification of Significant Communications via CellChat Applied to scRNA Data

To predict important ligand-receptor (L-R) interactions, intercellular communication networks were developed using ‘CellChat’ in label-free mode [15]. CellChatDB is a curated database of 2021 validated L-R complexes (informed by KEGG.db and primary literature), a constituent of cell-to-cell contact, ECM-receptor, and secreting interactions that has demonstrated immense utility in similar efforts. L-R interactions between computationally derived cell type pairs are modeled using the law of mass action, and communication probability (or strength) is quantified via network propagation to project expression counts onto validated protein-protein networks. Statistical significance is determined by a random permutation test, with group labels shuffled 100 times.

CellChat was run in the default configuration, with cell-cell communication groups derived from fewer than 10 cells excluded. CellChat’s extractEnrichedLR function was used to extract significant L-R pairs and related signaling genes for each of the obtained signaling pathways.

### 2.4. Identification of Significant L-R Interactions via Relative Crosstalk Scores Derived from Bulk RNA Data

An additional source of significant communication pathways within the melanoma interactome was derived from our previously published data, which utilized relative crosstalk (RC) scores calculated from the SKCM-TCGA bulk RNA dataset [6,16].

In brief, the ESTIMATE algorithm [17] was used to predict tumor purities, defined as the proportion of tumor cells in each tissue sample. Gene expression in the tumor and stromal (nontumor) compartments was modeled as follows:

$$e_{bulk,i}=p_{i}{\bar{e}}_{T}+\left(1-p_{i}\right){\bar{e}}_{S}$$

where:

- $e_{bulk,i}$ represents the bulk mRNA expression for a given gene in tumor sample *i*,
- $p_{i}{\bar{e}}_{T}$ is the average mRNA expression level in the tumor compartment, and
- $\left(1-p_{i}\right){\bar{e}}_{S}$ is the average mRNA expression level in the stromal compartment

Tumor and stroma compartment expression levels were obtained via negative least-squares regression, assuming consistent average compartment expression levels across tumor samples. This compartmental expression data was annotated with a curated set of 1,380 ligand-receptor pairs. Applying the Law of Mass Action, the molar concentration of a given ligand-receptor interaction complex can be modeled as follows:

$$\left[LR\right]=\left[L\right]\left[R\right]K_{D}^{-1}$$

Assuming that (1) inferred mRNA expression values serve as reasonable proxies for ligand and receptor concentrations, (2) ligand-receptor kinetics are uniform across samples, and (3) mass action principles are upheld, the RC score equation below was utilized. The numerator represents the ligand-receptor complex of interest, while the denominator accounts for all potential ligand-receptor interactions (or directionalities).

$$RC_{T,S}=\frac{\left({\bar{e}}_{L,T}{\bar{e}}_{R,S}\right)K_{D}^{-1}}{({\bar{e}}_{L,T}{\bar{e}}_{R,S})K_{D}^{-1}+({\bar{e}}_{L,T}{\bar{e}}_{R,T})K_{D}^{-1}+({\bar{e}}_{L,S}{\bar{e}}_{R,S})K_{D}^{-1}+\left({\bar{e}}_{L,S}{\bar{e}}_{R,T}\right)K_{D}^{-1}}$$

where:

- $RC_{T,S}$ represents the relative crosstalk score for a tumor-stroma ligand-receptor interaction,
- ${\bar{e}}_{L,T}{\bar{e}}_{R,S}$ represents the interaction of a tumor ligand with a stromal receptor,
- ${\bar{e}}_{L,T}{\bar{e}}_{R,T}$ represents the interaction of a tumor ligand with a tumor receptor,
- ${\bar{e}}_{L,S}{\bar{e}}_{R,S}$ represents the interaction of a stromal ligand with a stromal receptor, and
- ${\bar{e}}_{L,S}{\bar{e}}_{R,T}$ represents the interaction of a stromal ligand with a tumor receptor.

Simplifying further, the RC score for a specific tumor ligand and stromal receptor pair is calculated as:

$$RC_{T,S}=\frac{{\bar{e}}_{L,T}{\bar{e}}_{R,S}}{{\bar{e}}_{L,T}{\bar{e}}_{R,S}+{\bar{e}}_{L,T}{\bar{e}}_{R,T}+{\bar{e}}_{L,S}{\bar{e}}_{R,S}+{\bar{e}}_{L,S}{\bar{e}}_{R,T}}$$

This formulation provides a robust measure of tumor-stroma crosstalk, allowing for the identification of significant L-R interactions critical to the melanoma microenvironment.

### 2.5. Kaplan-Meier Survival Analysis of Consensus Regulatory Genes

For each regulatory gene, patient groups representing the top 50% and bottom 50%, by median gene expression were created using SKCM-TCGA bulk expression data. Differences in corresponding overall and disease-free survival between patient groups were then tested by Kaplan-Meier analysis. Log-rank *p*-values were adjusted for false discovery rate. Adjusted *p*-values < 0.05 were considered statistically significant.

## 3. Results

**Figure 1:**
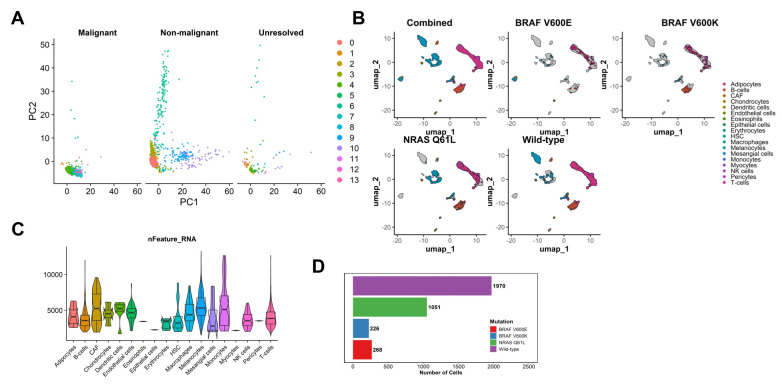
Raw data visualization. (**A**) Single-cell counts by driver mutation of tumor of origin. (**B**) Principal component analysis biplot of single cells by inferred malignancy status, annotated by Seurat cluster. (**C**) Uniform manifold approximation and projection biplot of single cells by driver mutation of tumor of origin, annotated by inferred cell type. (**D**) Feature counts by inferred cell type.

A total of 3515 single cells from fourteen (14) patients with available genotyping information were analyzed: 1970 from wild-type melanomas, 1051 from NRAS Q61L melanomas, 226 from BRAF V600K melanomas, and 268 from BRAF V600E melanomas (Figure 1A). The first two principal components, grouped by malignancy status and annotated by Seurat cluster, are illustrated in Figure 1B. Dimensionality reduction revealed areas of clear clustering based on cell type, especially between T-cells, B-cells, and melanocytes. Eosinophils, epithelial cells, myocytes, and pericytes were sparsely represented in our dataset (Figure 1C,D).

### 3.1. Generation of Co-Expression Networks

**Figure 2:**
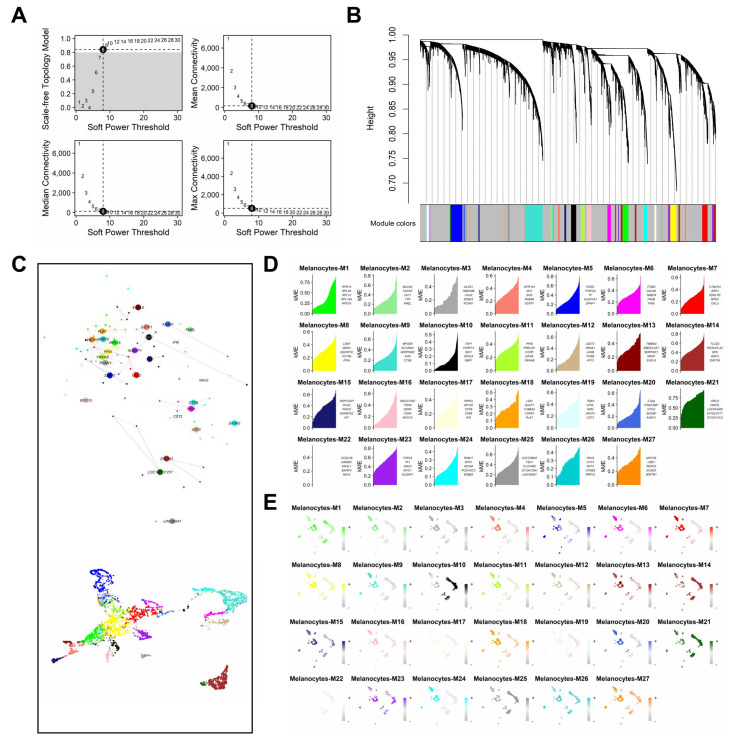
hdWGCNA pipeline: generation and analysis of weighted gene co-expression network modules. (**A**) Plots of scale-free topology (**top left**), mean connectivity (**top right**), median connectivity (**bottom left**), and max connectivity (**bottom right**) as a function of soft power threshold. (**B**) Module dendrogram, where grey coloration indicates an unresolved module. (**C**) Modules generated from melanocytes with corresponding top 5 hub genes, ranked by kME value. (**D**) Unified network plot comprised of hub genes as nodes and edge as relationships (**top**) and UMAP of the network topological overlap matrix (**bottom**). (**E**) Module feature plots of hub genes scores, derived by UCell algorithm.

Application of hdWGCNA to the global single-cell population generated twenty-seven (27) gene modules (Figure 2A–D). From these, a total of 270 hub genes were obtained, with kME values ranging from 0.26 to 0.93 (median kME = 0.54).

**Figure 3:**
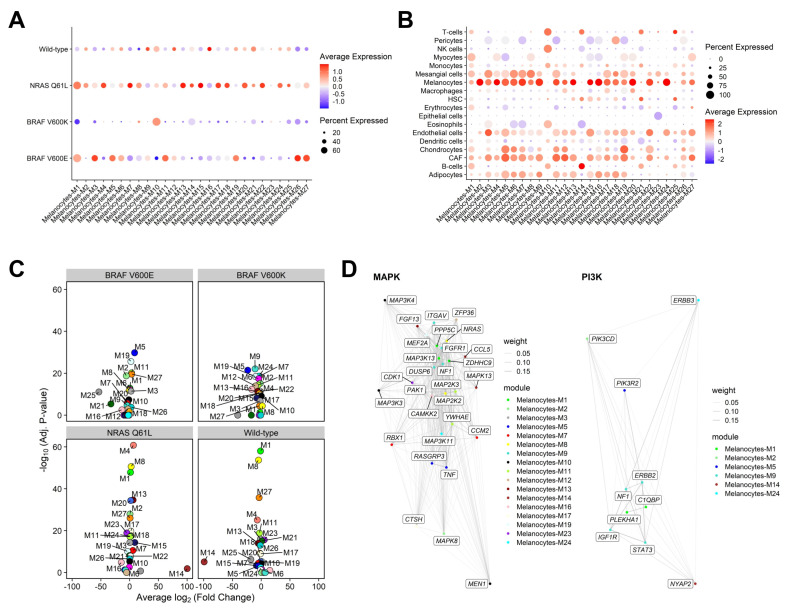
(**A**) Dot plot of MEs by driver mutation. (**B**) Dot plot of MEs by inferred cell type. (**C**) Differential module eigengene analysis, grouped by driver mutation. (**D**) MAPK (**left**) and PI3K (**right**) pathways annotated by gene module membership.

Among modules with ≥40% expression, wild-type melanomas were most enriched for module 16 (top 3 hub genes, by kME: *MGC27345*, *TSFM*, *GDNF*) and moderately downregulated for module 26. NRAS Q61L melanomas were most enriched for modules 7 (*C19orf10*, *ARF4*, *KDELR2*), 13 (*TIMM50*, *ZBED3*-*AS1*, *SERPINF1*), and 15 (*RAP1GAP*, *OCA2*, *PAICS*). BRAF V600K melanomas were downregulated for module 1 (*RPS15*, *RPL29*, *RPL10*). BRAF V600E melanomas were most enriched for modules 3 (*ALAS1*, *HMG20B*, *CALR*), 26 (*PAN3*, *CCT2*, *BTF3*), and 27 (*MYO1B*, *USE1*, *REXO2*), and moderately downregulated for module 7 (Figure 3A, B). Analysis of differential MEs revealed module 14 (*TLCD2*, *PRICKLE2*-*AS3*, and *SPN*) was significantly upregulated by NRAS Q61L melanomas (log2fc = 100.2, adjusted *p* = 1.32 × 10^−2^) and downregulated by BRAF V600E melanomas (log2fc = −206.3, adjusted *p* = 3.51 × 10^−14^) (Figure 3C).

Module membership of genes belonging to the mitogen-activated protein kinase (MAPK) and phosphatidylinositol 3-kinase (PI3K) and pathways is shown in Figure 3D.

### 3.2. Predicted Ligand-Receptor Interactions

To predict significant components of the melanoma L-R interactome, we applied CellChat to the complete single-cell population, identifying a total of 407 signaling genes involved in significant L-R interactions.

**Figure 4:**
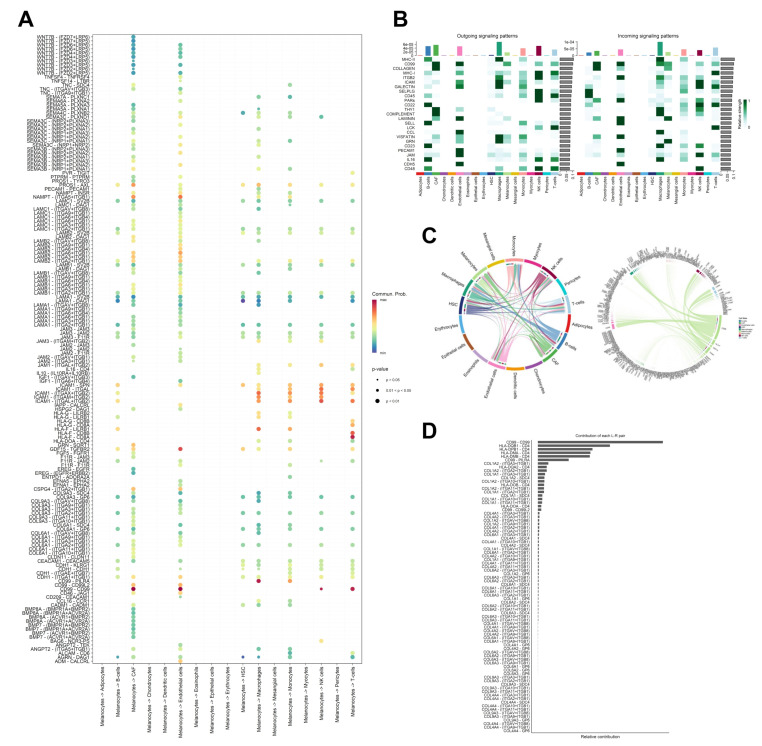
CellChat pipeline: significant L-R interactions and pathways. (**A**) Bubble plot of significant interactions (secreted signaling, cell-cell contact, and ECM-receptor signaling) from melanocytes to all other cell types. (**B**) Heatmap of highest-contributing outgoing (**left**) and incoming (**right**) signaling pathways. (**C**) Chord diagrams of significant interactions between all cell types (**left**) and from melanocytes on all other cell types (**right**). (**D**) Relative contribution of the top L-R interactions.

Assessment of interactions between melanocytes (source) and other cell types (targets) revealed that, most significantly, melanocytes communicated with CAFs (*p* < 0.01), endothelial cells (*p* < 0.01), NK cells (*p* < 0.05), and T-cells (*p* < 0.01) via CD99-CD99 interactions, as well as with macrophages via CD99-CD99L2 (*p* < 0.01). Additionally, strong signaling from melanocytes was detected with endothelial cells via GDF15-TGFBR2 (*p* < 0.01), T-cells via HLA-F-CD8A (*p* < 0.01), and NK cells via ICAM1-(ITGAL+ITGB2) (*p* < 0.01) (Figure 4A).

Overall, the top 3 most active signaling pathways were MHC-II, CD99, and Collagen-receptor signaling, and the top 3 most contributory L-R pairs were CD99-CD99, HLA-DQB1-CD4, and HLA-DPB1-CD4 (Figure 4B). Assessment of the signaling interaction network highlighted the central roles of macrophages, CAFs, and B-cells, as well as a prominent reliance of melanocytes on *CD99*, *HLA-F*, *GDF15*, and *ICAM1* (Figure 4C). Overall, CD99-CD99, HLA-DQB1-CD4, and HLA-DPB1-CD4 interactions demonstrated the highest contributions across the signaling network (Figure 4D).

**Figure 5:**
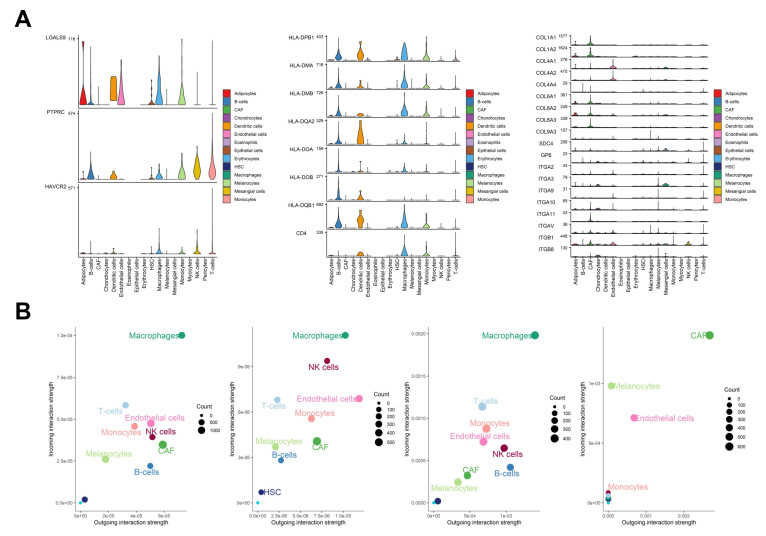
(**A**) Violin plot of signaling genes related to the galectin (**left**), MHC-II (**middle**), and collagen (**right**) pathways. (**B**) Scatterplots displaying dominant sender and receiver cell types for all pathways, secreted signaling, cell-cell contact, and ECM-receptor signaling.

To understand which cell types are dominant senders (i.e., cell types sending signals) and which are dominant receivers (i.e., cell types receiving signals), with respect to each L-R interaction analyzed, incoming and outgoing interactions strengths (weights) were calculated in the context of all pathways, secreted signaling, cell-to-cell contact, and ECM-receptor interactions. Respectively, the galectin signaling pathway emerged as the greatest contributor to secreted signaling, MHC-II signaling to cell-to-cell contact, and collagen signaling to ECM-receptor interactions (Figure 5A). Additionally, macrophages displayed the most robust overall incoming and outgoing interaction strength among immune cells (Figure 5B). CAFs showed the strongest incoming and outgoing ECM-receptor interaction strengths. Melanocytes had relatively poor outgoing interaction strength and middling incoming interaction strength. Moreover, NK cells demonstrated uniquely strong secreted signaling.

From our prior investigation, we extracted an additional set of eighty-five (85) genes. These genes encoded for L-R pairs with high relative crosstalk scores (top 15) along each of the tumor-to-stroma, stroma-to-tumor, tumor-to-tumor, and stroma-to-stroma signaling axes in a mixed sample of primary and metastatic melanoma.

### 3.3. Testing the Prognostic Value of Each Consensus Gene

**Figure 6:**
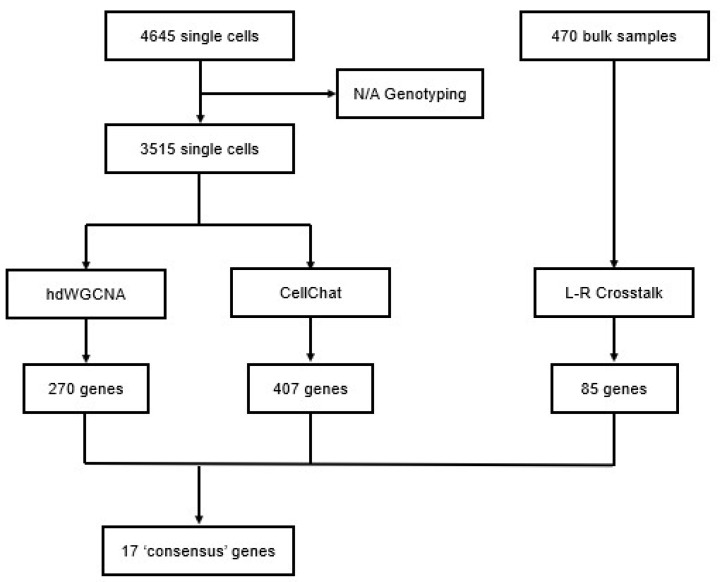
Overview of consensus gene selection.

A total of seventeen (17) unique genes were shared by at least two sets of genes resolved from the weighted gene co-expression networks, CellChat communications, and L-R RC scores (Table 1) (Figure 6).

**Figure 7:**
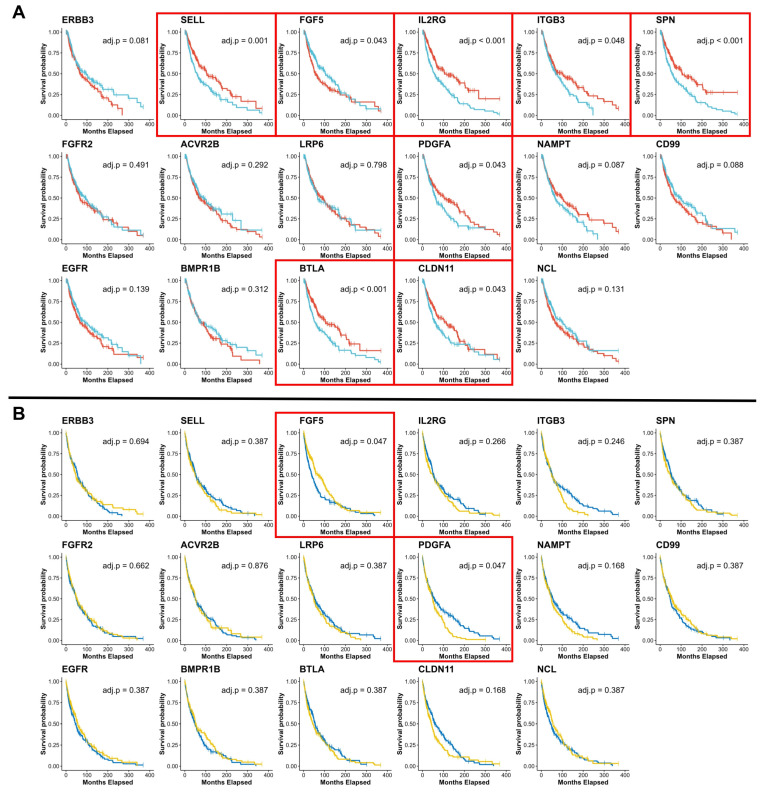
Kaplan-Meier survival analysis. (**A**) Comparison of overall survival (months) between patients with high or above-median (red) vs. low or below-median (light blue) consensus gene expression. (**B**) Comparison of disease-free survival (months) between patients with high or above-median (blue) vs. low or below-median (yellow) consensus gene expression. Red box indicates FDR-adjusted log-rank *p*-value < 0.05.

KM analysis revealed increased overall survival in patients with higher melanoma expression of *SELL* (median 105.0 months vs. 58.0 months, FDR-adjusted *p* = 1.37 × 10^−3^), *BTLA* (median 107.1 months vs. 53.9 months, FDR-adjusted *p* = 4.26 × 10^−4^), *IL2RG* (median 107.3 months vs. 55.5 months, FDR-adjusted *p* = 4.74 × 10^−5^), *PDGFA* (median 103.1 months vs. 62.8 months, FDR-adjusted *p* = 4.26 × 10^−2^), *CLDN11* (median 112.5 months vs. 61.1 months, FDR-adjusted *p* = 4.32 × 10^−2^), *ITGB3* (median 103.0 months vs. 69.0 months, FDR-adjusted *p* = 4.77 × 10^−2^), and *SPN* (median 107.1 months vs. 58.5 months, FDR-adjusted *p* = 4.26 × 10^−4^). Conversely, lower expression of *FGF5* was correlated with increased overall survival (median 61.0 months vs. 105.0, FDR-adjusted *p* = 4.32 × 10^−2^). Expression levels of *ERBB3*, *FGFR2*, *EGFR*, *ACVR2B*, *BMPR1B*, *LRP6*, *NAMPT*, *NCL*, and *CD99* were not significantly associated with overall survival (Table 2) (Figure 7A).

Better disease-free survival probability was reported in patients with higher expression of *PDGFA* (median 55.5 months vs. 48.0 months, FDR-adjusted *p* = 4.73 × 10^−2^) and in patients with lower expression of *FGF5* (median 66.0 months vs. 37.9 months, FDR-adjusted *p* = 4.73 × 10^−2^). None of *SELL*, *BTLA*, *IL2RG*, *CLDN11*, *ITGB3*, and *SPN* proffered a disease-free survival benefit (Table 2) (Figure 7B).

### 3.4. Mapping Expression Patterns of Prognostic Consensus Genes in Melanoma

**Figure 8:**
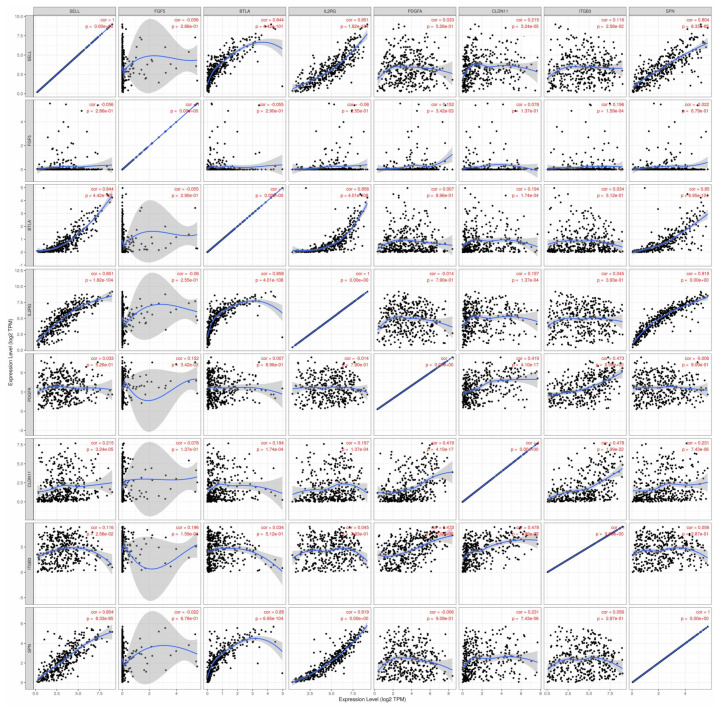
Spearman correlation analysis of consensus genes.

Using SKCM-TCGA RNA data, strong positive (defined as Spearman’s rho [ρ] > 0.8) correlations were observed between the expression levels of *SELL* and *BTLA* (ρ = 0.844, *p* = 4.42× 10^−101^); levels of *SELL* and *IL2RG* (ρ = 0.851, *p* = 1.82 × 10^−104^); levels of *SELL* and *SPN* (ρ = 0.804, *p* = 8.33 × 10^−85^); levels of *BLTA* and *IL2RG* (ρ = 0.858, *p* = 4.01 × 10^−108^); levels of *BLTA* and *SPN* (ρ = 0.850, *p* = 6.95 × 10^−104^); and levels of *IL2RG* and *SPN* (ρ = 0.919, *p* ~ 0) (Figure 8).

**Figure 9:**
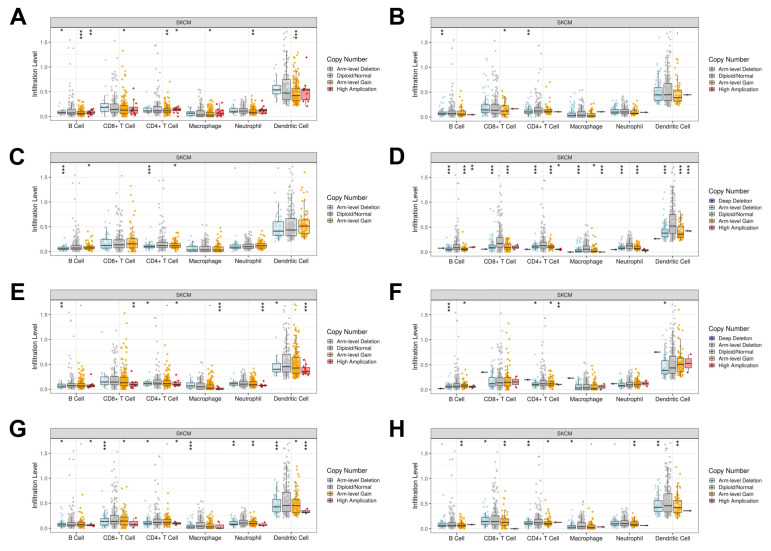
Association between levels of immune cell infiltration and copy number alterations of (**A**) *SELL*, (**B**) *FGF5*, (**C**) *BTLA*, (**D**) *IL2RG*, (**E**) *PDGFA*, (**F**) *CLDN11*, (**G**) *ITGB3*, and (**H**) *SPN*. *: *p* < 0.05; **: *p* < 0.01; ***: *p* < 0.001.

Understanding the impact of copy number alterations on immune cell infiltration is crucial for identifying potential mechanisms of immune evasion in melanoma (Figure 9). Compared to tumors with diploid/normal copy number, levels of B cell and CD4+ T cell infiltration were reduced in tumor samples with arm-level deletion of *BTLA*, *CLDN11*, and *FGF5*. Tumors with arm-level deletion or arm-level gain of *IL2RG* were associated with decreased infiltration of B cells, CD8+ T cells, CD4+ T cells, macrophages, neutrophils, and dendritic cells (*p* < 0.001). Similarly, arm-level deletion and arm-level gain of *SPN* were predictive of lower infiltration levels of CD8+ T cells, CD4+ T cells, and dendritic cells. Arm-level deletion of ITGB3 was most correlated with decreased infiltration of CD8+ T cells, macrophages, and dendritic cells (*p* < 0.001); high amplification of *ITGB3* also correlated with decreased dendritic cell infiltration (*p* < 0.001). High amplification of *PDGFA* was significantly associated with lower infiltration levels of CD8+ T cells (*p* < 0.01), CD4+ T cells (*p* < 0.05), macrophages (*p* < 0.001), neutrophils (*p* < 0.001), and dendritic cells (*p* < 0.001). Arm-level gain of *SELL* correlated with significantly decreased infiltration of B cells (*p* < 0.001), CD8+ T cells (*p* < 0.05), CD4+ T cells (*p* < 0.01), neutrophils (*p* < 0.01), and dendritic cells (*p* < 0.001); high *SELL* amplification similarly correlated with decreased infiltration of B cell (*p* < 0.01), but correlated with significantly increased CD4+ T cell infiltration levels (*p* < 0.05).

**Figure 10:**
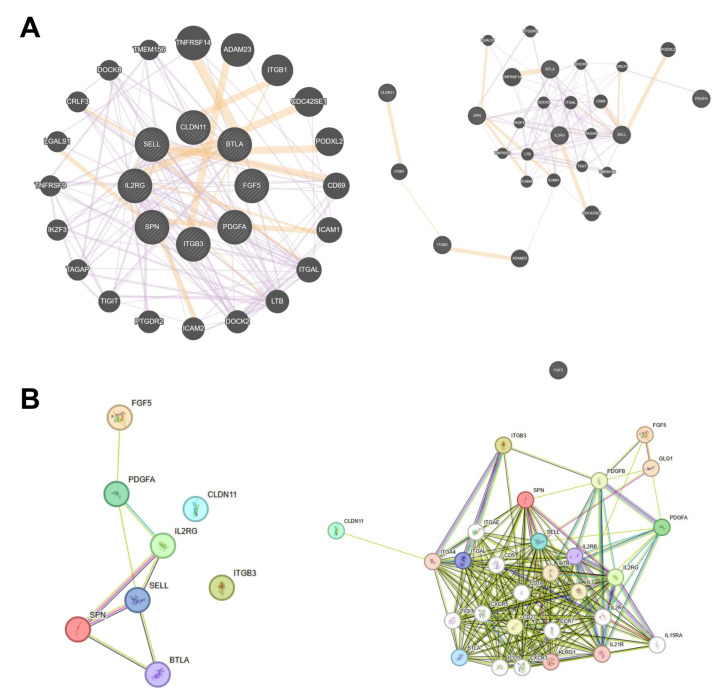
(**A**) GeneMANIA network of functional associations among consensus genes (**left**) and an alternate view (**right**). (**B**) StringDB protein-protein interaction networks of consensus genes (**left**) and expanded network (**right**).

Interconnectivity between consensus genes was further interrogated by StringDB (https://string-db.org/, accessed on 1 April 2024) and GeneMANIA (https://genemania.org/, accessed on 1 April 2024) (Figure 10A,B). The String protein-protein interaction network was found to have eight edges (expected edges = 1) (*p* = 8.62 × 10^−6^), with an average local clustering coefficient of 0.521, suggesting the inputted nodes are not random. An expanded String network of 28 nodes contained 234 edges (expected edges = 35) (*p* < 1.0 × 10^−16^), with an average local clustering coefficient of 0.802. GeneMANIA revealed network enrichment for cellular extravasation (FDR-adjusted *p* = 2.83 × 10^−6^, coverage = 6/56), leukocyte migration (FDR-adjusted *p* = 2.47 × 10^−5^, coverage = 8/281), and leukocyte cell-cell adhesion (FDR-adjusted *p* = 2.47 × 10^−5^, coverage = 8/274).

## 4. Discussion

In the present study, we present a picture of malignant melanoma as an active yet organized cancer. The results indicated that wild-type melanomas enriched genes network modules regulated by genes involved in mitochondrial function; NRAS Q61L melanomas were most enriched for modules regulated by C19orf10, ARF4, KDELR2; BRAF V600K melanomas downregulated modules regulated by ribosomal proteins; and BRAF V600E melanomas were most enriched for modules regulated by processing and trafficking proteins. Tumor-associated macrophages expectedly played a dominant role in outgoing and incoming communications, confirming what is understood in other cancers [18,19].

We also distinguished a subset of key signaling genes with apparent significance in melanoma survival outcomes. Interestingly, correlation and network analyses suggested these genes (1) are interconnected, (2) enrich their environments for cellular extravasation, leukocyte migration, and cell-cell adhesion, and (3) are predictive of differential immune infiltrates by somatic copy number alteration.

*SELL* encodes a cell surface adhesion molecule that recognizes sialylated carbohydrate groups. Higher expression of *SELL* was associated with better survival outcomes in basal, Her2 +, and luminal B subtypes of breast cancer [20]. Ji and colleagues had also described *SELL* as a hub gene in their use of TCGA expression data but did not realize any effect on survival (albeit using a Cox regression approach) [21].

*BTLA* encodes for a protein that functions as an immune checkpoint receptor to regulate immune responses [22]. Elevated levels of *BTLA* in melanoma have been previously associated with increased immune filtration levels (namely CD8+ T cells) and improved prognosis [23]. *BTLA* expression has been specifically associated with improved clinical responses in patients undergoing adoptive T-cell therapy for metastatic melanoma [24]. However, other research indicates that *BTLA* can inhibit tumor-specific CD8+ T cells, potentially compromising anti-tumor immunity [25].

*IL2RG* encodes for a subunit of the interleukin-2 receptor complex, which regulates T-cell function, was previously linked to poorer prognosis in gastric cancer [26], and IL2Rγ/JAK3 signaling has been shown to contribute to pancreatic cancer proliferation in vivo [27]. The rapid in vitro loss of IL2Rγ expression in cultured cells has posed a significant challenge to fully elucidating the intricacies of the IL2Rγ signaling pathway, restricting further insights into its functional dynamics [28].

High expression of *PDGFA* predicts poor prognosis of esophageal squamous cell carcinoma [29]. *PDGFA*-positive immunostaining had a higher likelihood of the risk of death (hazard ratio = 2.907, *p* = 0.016) [30]. A gene expression analysis found *PDGFA* to have significantly higher expression levels in esophageal cancer tissues compared to corresponding normal samples [31]. This upregulation suggests a potential role for *PDGFA* in tumor progression and pathology.

Several claudin genes (including *CLDN11*) are downregulated in colon adenocarcinoma [32]; Li et al. previously reported increased metastasis and worse progression-free survival in colorectal cancer patients with methylated *CLDN11* [33]. Increased expression of *CLDN11* has also been linked to better overall breast cancer survival [34].

*ITGB3* is associated with melanoma progression and metastasis-promoting potential as its overexpression causes melanoma cells to transition from the radial growth phase to the vertical growth phase [35]. The expression of *ITGB3* RNA was linked with prolonged survival of melanoma patients, supporting its potential role as an effector in the metastasis suppressor function of NME1 [36]. Haqq et al. reported a significant increase in the transcription level of *ITGB3* in melanoma patients, showing a 3.147-fold change compared to normal skin tissue [37]. Thus, abnormal expression of *ITGB3* in melanoma may serve as a prognostic marker [38].

Elevated *SPN* expression hinders the clustering of tumor cells with T cells, which reduces the effectiveness of CD3 bispecific antibody-mediated tumor cell lysis [39]. However, *SPN* otherwise remains poorly described in the literature.

*FGF5* is characterized as a critical regulator of hair growth that promotes angiogenesis, differentiation, and cell proliferation [40]. Overexpression of *FGF5* may promote melanoma growth through increased activation of the MAPK pathway [41]. *FGF5* overexpression in melanoma cells suggests pro-tumorigenic functions with enhanced malignancy through paracrine effects [41,42].

Overall study limitations include low sample size (and thus total cell counts); restricted (by missing) genotyping for several tumors; use of a separate non-metastatic sample for assessing survival outcomes; noisiness inherent to single-cell analysis; and reliance on CellChat for cell-to-cell communication analysis, which may miss uncharacterized or context-specific interactions due to its dependence on predefined L-R pairs and static assumptions. Additionally, the computation of relative crosstalk scores in our previous analysis used mean expression across tumor and stromal compartments, which may discount heterogeneity in the latter. Nonetheless, the present study identifies a set of novel and testable gene candidates with potential therapeutic value in melanoma.

## 5. Conclusions

Our findings reveal the extensive diversity of the melanoma microenvironment, highlighting prominent contributions from tumor-associated macrophages and key L-R interactions along secreted signaling, ECM-receptor, and cell-cell contact signaling axes. Among master regulatory genes, we observed significant clinical relevance and functional network interconnectedness in *SELL*, *FGF5*, *BTLA*, *IL2RG*, *PDGFA*, *CLDN11*, *ITGB3*, and *SPN*.

The persistent challenge of primary and acquired resistance to current therapies underscores the need for novel, mechanistically driven therapeutic targets. The pathways and interactions identified in this study represent promising candidates for further investigation. Future efforts should prioritize in vivo validation and elucidate the mechanistic roles of these signalers within the melanoma interactome and their influence on tumor-immune dynamics. Such efforts are crucial for translating these insights into precision oncologic strategies to improve patient outcomes.

## Figures and Tables

**Table 1:** Overlapping gene set.

HUGO	Name	Chr	Function
*SELL*	Selectin L	Chr1	Leukocyte adhesion and migration
*NCL*	Nucleolin	Chr2	Ribosome biogenesis and nucleic acid binding
*ACVR2B*	Activin A Receptor Type 2B	Chr3	Signal transduction
*BTLA*	B and T Lymphocyte Attenuator	Chr3	Immune response regulation
*CLDN11*	Claudin 11	Chr3	Tight junction formation
*BMPR1B*	Bone Morphogenetic Protein Receptor Type 1B	Chr4	Bone and cartilage development
*FGF5*	Fibroblast Growth Factor 5	Chr4	Regulation of cell proliferation and differentiation
*EGFR*	Epidermal Growth Factor Receptor	Chr7	Cell proliferation and survival
*PDGFA*	Platelet-Derived Growth Factor Subunit A	Chr7	Cell growth, division, and angiogenesis
*NAMPT*	Nicotinamide Phosphoribosyltransferase	Chr7	NAD biosynthesis
*FGFR2*	Fibroblast Growth Factor Receptor 2	Chr10	Cell growth and differentiation
*ERBB3*	Erb-B2 Receptor Tyrosine Kinase 3	Chr12	Signal transduction
*LRP6*	Low-Density Lipoprotein Receptor-Related Protein 6	Chr12	Wnt signaling
*SPN*	Sialophorin	Chr16	Cell adhesion
*ITGB3*	Integrin Subunit Beta 3	Chr17	Cell adhesion and signal transduction
*IL2RG*	Interleukin 2 Receptor Subunit Gamma	ChrX	Cytokine signaling
*CD99*	CD99 Molecule	ChrX	Cell adhesion and migration

**Table 2:** Overall survival (OS) and disease-free survival (DFS) analyses of top and bottom 50% groups by expression level.

Gene	Median OS Months, High Expression Group	Median OS Months, Low Expression Group	Adjusted Log-Rank *p*-Value, OS	Median DFS Months, High Expression Group	Median DFS Months, Low Expression Group	Adjusted Log-Rank *p*-Value, DFS
*SELL*	105	58	**1.37 × 10^−3^**	51.5	48.2	3.87 × 10^−1^
*NCL*	66.4	103.2	1.31 × 10^−1^	44.6	55.5	3.87 × 10^−1^
*ACVR2B*	68.1	92.9	2.92 × 10^−1^	51.1	49.4	8.76 × 10^−1^
*BTLA*	107.1	53.9	**4.26** × 10^−4^	55.4	43	3.87 × 10^−1^
*CLDN11*	112.5	61.1	**4.32** × 10^−2^	59	42.4	1.68 × 10^−1^
*BMPR1B*	78.9	80.6	3.12 × 10^−1^	48.6	51.4	3.87 × 10^−1^
*FGF5*	61.0	105.0	**4.32** × 10^−2^	37.9	66.0	**4.73** × 10^−2^
*EGFR*	68	89.1	1.39 × 10^−1^	46.7	55.8	3.87 × 10^−1^
*PDGFA*	103.1	62.8	**4.26** × 10^−2^	55.5	48	**4.73** × 10^−2^
*NAMPT*	103	66	8.71 × 10^−2^	55.5	48	1.68 × 10^−1^
*FGFR2*	66.4	92.9	4.91 × 10^−1^	49.2	52.1	6.62 × 10^−1^
*ERBB3*	68.1	96.2	8.11 × 10^−2^	55.5	46.7	6.94 × 10^−1^
*LRP6*	89.1	66.6	7.98 × 10^−1^	52.1	48	3.87 × 10^−1^
*SPN*	107.1	58.5	**4.26** × 10^−4^	53.1	48.2	3.87 × 10^−1^
*ITGB3*	103	69	**4.77** × 10^−2^	51.5	48.2	2.46 × 10^−1^
*IL2RG*	107.3	55.5	**4.74** × 10^−5^	51.5	49.2	2.66 × 10^−1^
*CD99*	66.6	96.2	8.85 × 10^−2^	48.0	56.8	3.87 × 10^−1^

*p*-values < 0.05 bolded.

## Data Availability

The datasets underlying this study are publicly accessible. The single-cell RNA data can be found at the Broad Single Cell Portal (singlecell.broadinstitute.org/single_cell/study/SCP11). The bulk RNA data can be found at the Broad GDAC Firehose (https://gdac.broadinstitute.org/).
